# Supplementary material for: Learning to detect AI texts and learning the limits
Source: PLoS One. 2025 Oct 15;20(10):e0333007. doi: 10.1371/journal.pone.0333007 (PMC12527182; doi:10.1371/journal.pone.0333007)
Supplement: S2 File — All data and scripts used in the study, some additional descriptive statistics, scalable formate figures. (ZIP) [file pone.0333007.s002.tgz › data and scripts/OSF/scripts/analysis/Dependencies/GLS2_distance_vs_Reaction_time_log.pdf]

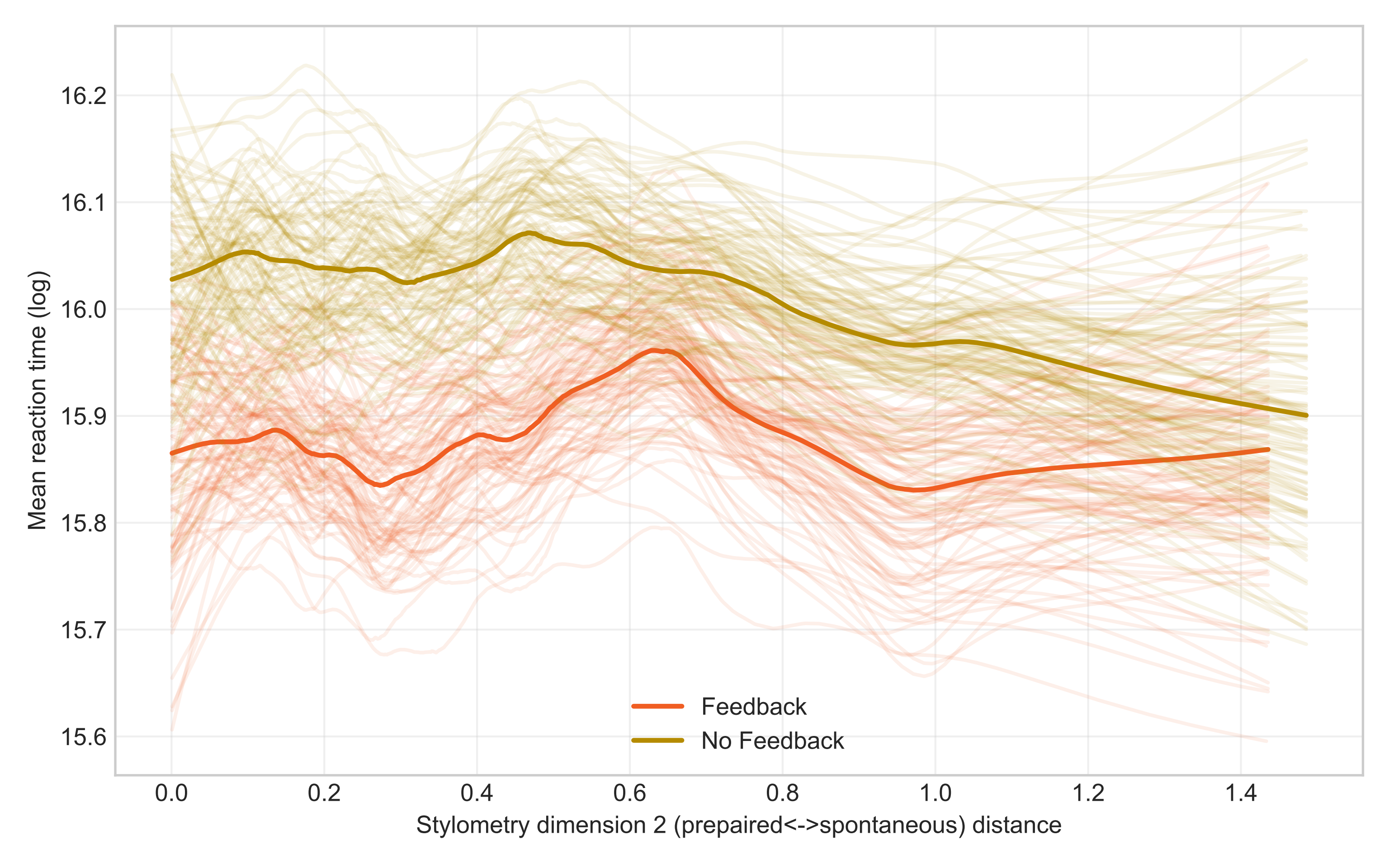

16.2
16.1
16.0
15.9
15.8
15.7
15.6
Mean reaction time (log)
0.0
0.2
0.4
0.6
0.8
1.0
1.2
1.4
Stylometry dimension 2 (prepaired<->spontaneous) distance
Feedback
No Feedback
